# Posture Control—Human-Inspired Approaches for Humanoid Robot Benchmarking: Conceptualizing Tests, Protocols and Analyses

**DOI:** 10.3389/fnbot.2018.00021

**Published:** 2018-05-07

**Authors:** Thomas Mergner, Vittorio Lippi

**Affiliations:** Department of Neurology, University Clinics of Freiburg, Freiburg, Germany

**Keywords:** humanoid robots, sensorimotor system, posture control, human-like versatility and robustness, benchmarking

## Abstract

Posture control is indispensable for both humans and humanoid robots, which becomes especially evident when performing sensorimotor tasks such as moving on compliant terrain or interacting with the environment. Posture control is therefore targeted in recent proposals of robot benchmarking in order to advance their development. This Methods article suggests corresponding robot tests of standing balance, drawing inspirations from the human sensorimotor system and presenting examples from robot experiments. To account for a considerable technical and algorithmic diversity among robots, we focus in our tests on basic posture control mechanisms, which provide humans with an impressive postural versatility and robustness. Specifically, we focus on the mechanically challenging balancing of the whole body above the feet in the sagittal plane around the ankle joints in concert with the upper body balancing around the hip joints. The suggested tests target three key issues of human balancing, which appear equally relevant for humanoid bipeds: (1) four basic physical disturbances (support surface (SS) tilt and translation, field and contact forces) may affect the balancing in any given degree of freedom (DoF). Targeting these disturbances allows us to abstract from the manifold of possible behavioral tasks. (2) Posture control interacts in a conflict-free way with the control of voluntary movements for undisturbed movement execution, both with “reactive” balancing of *external* disturbances and “proactive” balancing of *self-produced* disturbances from the voluntary movements. Our proposals therefore target both types of disturbances and their superposition. (3) Relevant for both versatility and robustness of the control, linkages between the posture control mechanisms across DoFs provide their functional cooperation and coordination at will and on functional demands. The suggested tests therefore include ankle-hip coordination. Suggested benchmarking criteria build on the evoked sway magnitude, normalized to robot weight and Center of mass (COM) height, in relation to reference ranges that remain to be established. The references may include human likeness features. The proposed benchmarking concept may in principle also be applied to wearable robots, where a human user may command movements, but may not be aware of the additionally required postural control, which then needs to be implemented into the robot.

## Introduction

Considerable progress in the sensorimotor skills of humanoid robots has been made over the recent years, such as in bipedal walking (Vukobratović and Borovac, 2004; Clever and Mombaur, 2017). Despite this progress, the human sensorimotor abilities still represent the “gold standard” for the humanoid robots (Nori et al., 2014; Torricelli et al., 2015, 2016). Currently, the robotics community is taking an important step towards developing objective standards for these skills^1^. An aim is to define benchmarks which allow for objective comparisons among robots and to thereby foster their progress. The benchmark tests may address in some form or other human-likeness of the robot’s performance (Torricelli et al., 2016). Furthermore, they address posture control as an issue to be tested in addition to movement performance. Posture control is typically required, for example, when a robot tries to maintain balance while walking across rough terrain or when it needs to compensate the gravitational ankle torque during a voluntary body lean. Generally, the ability of posture control can also be tested separately from movement control by applying external disturbance such as a push against the body while standing. In this Methods article, we develop a concept for benchmark tests of posture control in humanoid robots. The concept addresses the generic principles underlying the human posture control features versatility and failsafe robustness. We hold that these features are based on simple basic mechanisms by which, during phylogenetic development over millions of years, even primitive animal species have learned to use and to combine the solutions needed to deal with the physics of the terrestrial environment. Elaborating on their functional basis, we try to make them testable in humanoid robots for benchmarking. Our hypothesis is that providing the robots with human-like versatility and fail-safe robustness in their sensorimotor control will help them to perform better in *complex sensorimotor scenarios* (compare^2^).

To introduce our hypothesis, we will describe further below some basic sensorimotor mechanisms that we think are underlying the human versatility and fail-safe robustness abilities. We do not claim that these mechanisms are the only possible or ultimately best prerequisites for these abilities. Rather, our aim is to introduce some basic principles that shape the human sensorimotor and postural control and by this also the consequent human versatility and fail-safe robustness abilities. Versatility here is taken to mean that a standing human may involve in reaching with a hand, for example, also the torso and leg segments, thus involving either the hips joints or the ankle joints respectively, or some combination thereof. The choice for using hip and/or ankle joints provides some robustness in case that involvement of one or the other of these joints is not possible or falls short. This example shows one of several interrelations in the human sensorimotor system, which overall provide not only conflict-free interactions between its constituents, but also synergy effects and other benefits. Another example in the human sensorimotor systems is the causal chain of (*a*) the need to tolerate biological feedback times delays >100 ms, which (*b*) is achieved to a large degree by using a low loop gain for controlling the human actuation that, being force controlled, in turn shows (*c*) a soft mechanical compliance and in many situations (*d*) a low energy consumption (see Mergner and Peterka, 2017). Notably, each of the human solutions *a-d* taken alone may not reach optimality in terms of a specific cost function, but in view of their interrelation may represent “good enough” solutions (see also Loeb, 2012).

Interrelations also exist between posture control and voluntary movements in the form that posture control “proactively” compensates the self-produced disturbances arising from own motor activities such as the gravitational torque from a body lean in the ankle joints—this in addition to the “reactive” compensation of *external* disturbances, e.g., from an external push against the body that perturbs standing or walking balance (Mergner, 2010). Both disturbance compensations are needed to allow execution of poses and movements in the commanded (i.e., undisturbed) form. They involve posture control centers in the *extrapyramidal* *s*ystem (EPS; comprising basal ganglia, cerebellum, brainstem centers and the cortical supplementary motor areas). The functionality of the EPS is closely linked to that of the movement commanding “pyramidal system” in the cortical centers with projections to the brainstem and spinal cord. EPS impairments tend to severely affect sensorimotor control, as witnessed by a variety of motor impairments in neurological patients (Bastian, 1997; Visser and Bloem, 2005). Proactive disturbance compensation is predictive (feed forward) and therefore considered as advantageous compared to reactive (sensory feedback) compensation in terms of lower noise and shorter time delays, which is supposed to yield improved control stability and motor performance (Wolpert and Flanagan, 2001). The following article addresses for the testing of humanoid robots both proactive and reactive scenarios as well as their superposition.

The testing of humanoid robots for human-like versatility and robustness is eased if the robots use *torque-controlled actuation* as humans do. This would facilitate direct robot-human comparisons. More importantly, torque-controlled robots represent the current state of the art for “real world” applications. Advantages of the torque control are, for example, reduced damage when falling or when interacting or colliding with the environment, and also a better acceptance by humans when directly (physically) interacting with them. The robot tests suggested in the following are in principle, however, not specific for force-controlled actuation in that they address human-like versatility and fail-safe robustness as a general functional rather than mechanical property of the human system.

With the focus on human-like versatility and fail-safe robustness, our approach differs from recent concepts on benchmarking of robot walking and posture control, which are more general and list numerous performance tests and metrics (e.g., Torricelli et al., 2015). Despite similarities in the details of the suggested tests, a relevant difference here is that we rest our focus on basic principles of controlling the posture of a segmented biped under the premise of versatility and robustness. But we conceive that the tests and metrics first have to be discussed in this field, before certain concepts are finally favored and realized in a form outlined in Torricelli et al. (2015). We justify our approach with the problems that a robot benchmarking is facing when it is confronted, as expected, with many different control methods, mechanical constructions, and targeted applications of the robots, and with differences between testing procedures across the performing laboratories. Addressing basic control issues in our concept will help to abstract from these diversities. In our approach, we define as human-like postural versatility and fail-safe robustness the property and ability of the human sensorimotor system that enables humans to cope reactively with external disturbances and proactively with the self-produced disturbances in a flexible, yet efficient way by exploiting kinematic and kinetic inter-segmental interaction effects. Specifically, our approach considers the mechanically highly relevant interaction between whole body balancing in the ankle joints and upper body balancing in the hip joints.

With this goal, this article gives next a brief overview on basic issues of the human postural control system with a focus on the fundamental principles that are underlying the human versatility and fail-safe robustness. The following chapter then explains the basic methods we are suggesting for quantitatively describing human postural responses, before reporting then our attempts to implement human control principles in our bio-inspired humanoids and presenting examples of the suggested benchmark tests in our robots. In the following we consider quantification and metrics of benchmark results, before we finally discuss in the last chapter the usefulness of the human-inspired experiments and point out that testing human sensorimotor concepts in robots may provide beneficial impulses for both the human and robot posture control research.

## Basic Aspects of Human Posture Control

### Shaping of Postural Control by the Terrestrial Force Environment

**Figure 1:**
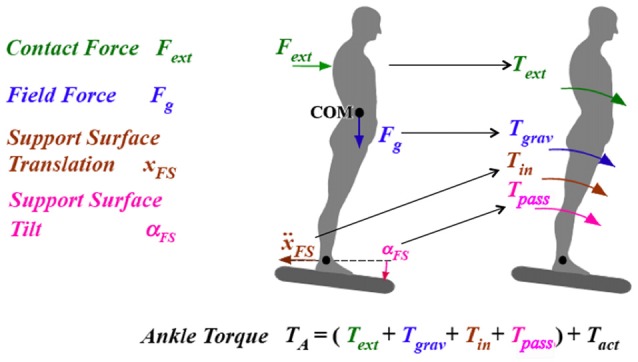
Single inverted pendulum (SIP) scenario of posture control in the body sagittal plane. Shown are the four basic disturbances (on the left) with impact on the ankle joints in terms of disturbing torques, as indicated by the arrows on the right side. *T_A_*, total ankle torque; *T_act_*, commanded active torque; *T_ext_*, external torque from contact force; *T_grav_* from field force gravity; *T_in_* from inertial effect upon foot in space translational acceleration (*Ẍ_FS_*; *T_pass_*, from passive stiffness of muscles and connective tissues.

Under terrestrial conditions, humans and humanoids are in a force environment where gravity plays an important role; in general, gravity compensation accounts for most of the joint torque produced during balancing tasks (see Zebenay et al., 2015 for a comparison in a humanoid biped with human-inspired posture control between the torques produced by different external effects). Therefore, it is not surprising that in humans the vestibular sensory system is one of the first to become behaviorally evident during human ontogenesis. Based mainly on vestibular signals, newborns soon learn to first bring the head and later the trunk upright for perceptual orientation in space and for interaction and communication with the world. Humanoids must also take gravity into account in most tasks. Other field forces need to be detected and counteracted as well, such as centrifugal forces in passive transport. Such forces must be compensated for in order to allow undisturbed execution of voluntary poses and movements of the body and its segments. This is schematically illustrated in Figure 1 for a human subject who tries to maintain the body upright or to move it into a desired orientation in space. Here, the subject is controlling the ankle joint torque in the body’s sagittal plane (biomechanically, a single inverted pendulum, SIP, scenario) against the four relevant external disturbances. These are, in addition to the field forces, the contact forces (e.g., from a push against the body) and the dynamic joint impacts associated with support surface (SS) rotation and translation. *Posture control refers in the following to the compensation of these joint impacts, independently of whether they are self-produced (such as the gravitational ankle torque with an active body lean) or external, and whether they are occurring during a held body pose (e.g., upright stance) or a movement*. The disturbance compensation allows humans to maintain a pose or to execute a movement as it is commanded in Figure 1 by the active torque *T*_act_. For compensation, the disturbances are estimated on the basis of sensory information or by learned predictions of the sensory disturbance estimates.

### Sensory Estimations of the Four Basic Disturbances and Their Predictions

In addition to the vestibular signals, other sensory signals such as those for joint angle and angular velocity and joint torque are used for human posture control (more in Mergner and Peterka, 2017). Visual information may additionally be used to improve the other sensory signals with respect to noise and accuracy, and it may partially serve as a substitute for loss in some sensory functions. The four external disturbances in the SIP scenario of Figure 1 can be estimated by combining several of these sensory signals (Mergner, 2010). Humans may learn from repeated presentations of external disturbances to predict them and to fuse predicted and sensor-derived disturbance estimates, as has been successfully mimicked in tests using a human-inspired robot (Mergner, 2010). In these tests, it was also assumed that commanding voluntary movements is associated with prediction of sensory disturbance estimates for the self-produced joint impacts. Dealing with disturbance estimates rather than with the underlying manifold of sensory transducer signals greatly reduces processing complexity for sensorimotor control, and it also appears parsimonious for the linkages between sensorimotor control, perception and cognition.

### Analytical Description of the Human Postural Control

A prominent feature of human postural control is the *sensory re-weighting phenomenon*. It consists of a basically stereotype and continuous adjustments of the postural responses to the environmental and test conditions (unless stance stability requires a step). These adjustments can be described as resulting from changes in the weighting factors of sensory feedback loops, as it has been analyzed in human data obtained for a SIP scenario using visual, vestibular, and joint angle sensory information in a simplified linear model of the human sway responses to moderate SS tilts in the body’s sagittal plane (“*i*ndependent sensory *c*hannel,” IC, model; Peterka, 2002). Adjustments to a stepwise increase in surface rotation magnitude were captured using a family of gain curves in terms of a describing function. The identified time delays were in the order of 150–200 ms, and the loop gain was slightly above the minimum to resist gravity. Comparable system identification procedures were later applied to the biomechanically more complex scenario of lower and upper body responses to SS tilt in the frontal plane (Goodworth and Peterka, 2010). However, the number of model parameters needed to describe the control on the basis of the experimental data clearly increased, while confidence in parameter identification and in the attribution to specific physiological processes decreased, which limits the use of a corresponding model for clinical purposes.

### Heuristic Model of Human Posture Control

The four disturbances shown in Figure 1 and their estimation and prediction mechanisms are the basis of the *disturbance estimation and compensation* (DEC) model (Mergner, 2010), which can be viewed as building upon the IC in that it is able to describe the same set of experimental data. However, the DEC model describes the data with one set of control parameters. In addition, it can also describe responses to other stimuli that are typically used in posture control experiments such as pushes that impact the body. The overall resulting increase in versatility comes, however, at the expense of an increase in model complexity, which includes more sensory signals (e.g., velocity signals in the disturbance estimation channels) and nonlinear operators (threshold elements in these channels). This model entails, in addition, a qualitative step forward in robustness, in that it allows for a conflict-free superposition of two or more of the four disturbances at a time, be these external or self-produced. This improvement is made possible by combining the compensation of all four disturbances with the control of movement execution in one control mechanism (see Mergner, 2010). The control is realized as a servo loop controller for commanding the actuator to produce the force that is required to execute a desired movement or force. Superimposed on this force command are commands from the disturbance estimators for compensating the disturbance forces. Executed action then corresponds to the desired action to the extent to which disturbance compensation is complete (note that removing in Figure 1 the four disturbing torques makes the total ankle torque *T*_A_ equal to the commanded torque *T*_act_). This control principle appears to apply to the majority of the human skeletal joints and their degrees of freedom (DoFs) and, after implementing it in the following in modular control architecture, provides the basis for our concept of human versatility and robustness.

### Modular Control Architecture of DEC Modules

Human-like versatility and robustness profits from combining, in a flexible way, several joints in a task performance. A well-known example in posture control research is the involvement of hip movements when the balancing of stance in the ankle joints tends to become insufficient (McCollum et al., 1985). The involved sensory signals are distributed by coordinate transformations across the joints of the body, as described for the vestibular signals that arise in the head and are used to sense motion of the support base (Mergner and Rosemeier, 1998). Such sensory interrelations between body segments inspired the concept of a modular control architecture consisting of a net of interconnected DEC control modules, one for each DoF in the three planes of the human body (sagittal, frontal, horizontal; Lippi et al., 2013). Proof-of-principle tests in human-inspired robots were positive and, in addition, revealed functional emergencies such as an inter-segmental hip-ankle coordination (Hettich et al., 2014; Lippi and Mergner, 2017). In this architecture, a shift of activity from “ankle strategy” to “hip strategy” (see below) or, for example, from a pain-blocked knee to the neighboring joints when walking, occurs *per default*. This is here thought to represent the basis for the human robustness, while it is attributed mostly to versatility when performed proactively.

### Mutual Inspirations Between Robotics and Human Posture Control Research

Modeling and simulating human experimental results *per se* may have limited value in face of the high complexity of the human posture control and the many unknown factors such as sensor and actuation noise and inaccuracies. Implementing and testing the DEC model in human-inspired robots were performed for proof of principle and demonstration of “real world” robustness of the modeling results (Mergner et al., 2009; Mergner, 2010; Hettich et al., 2014; Lippi and Mergner, 2017). From this approach, progress for posture control of both humans and robots can be expected, and the same applies to testing alternative posture control concepts in the same robot (Alexandrov et al., 2017) and to testing of a given control concept on different robots (Ott et al., 2016). Using here human-derived criteria for robot-human and robot-robot benchmark comparisons represents a further variant of this issue. Generally, promising linkages between humanoid robotics and neuroscience are well recognized in both the robotics and neuroscience fields (e.g., Cheng et al., 2007).

## Conceptualizing Benchmarks Tests for Posture Control in Robots

Many posture control tests used in humans could also potentially be used in humanoid robots. Posture control in humans is most often tested under medical perspectives, e.g., for evaluation of a child’s development or of balance control deficits in diseases and with aging. These tests range from simple observations of standing balance with eyes closed vs. eyes open to sophisticated walking measures on a treadmill, under consideration of age, disease, etc. As for robots, their diversity with respect to control method, construction, etc. hampers attempts to globally apply the human benchmark ratings and the diagnostic criteria for malfunction. These considerations lead us here to mainly consider benchmark tests that challenge very basic postural skills such as the compensation of the four disturbances considered above (“Heuristic Model of Human Posture Control” section and Figure 1). Testing these skills may provide not only benchmark ratings, but also “diagnostic” hints in case of malfunction. As mentioned above, the tests we are suggesting are not restricted to compensation of *external* disturbances, but also cover “postural adjustments” occurring in association with voluntary movements. In contrast, the postural stabilization by “rescue steps” or by multi-contact body configurations such as to support the balancing with the hands, for example, are not considered here. The supportive effect of vision on human posture control will be considered only briefly and preliminarily. The robots’ adjustments to changes in body weight as they occur with lifting and carrying external loads are also left unaccounted for at present. Generally, we suggest performing all tests with the same set of control parameters to judge the robots’ automatic adjustment to the changes in the test condition. Furthermore, we suggest mainly covering the normal range of posture disturbances in the tests, which includes supportive use of the hip joints when balancing in the ankles joints, whereas “rescue reactions” to extreme challenges should remain unconsidered, such as how the rapid hip movements that typically occur when standing on a narrow beam renders the balancing in the ankle joints ineffective.

The approach we suggest here for posture control benchmarking aims to finally judge the performance of a given robot under the viewpoint of versatility and robustness in the sense that the robot would be able to deal with the relevant different types of disturbances or even their overlap, be they external or self-produced. This does not exclude, however, developing a robot that provides high performance in only one or a few tasks and less so in the others.

### Conceptualized Scenarios and Tests

**Figure 2:**
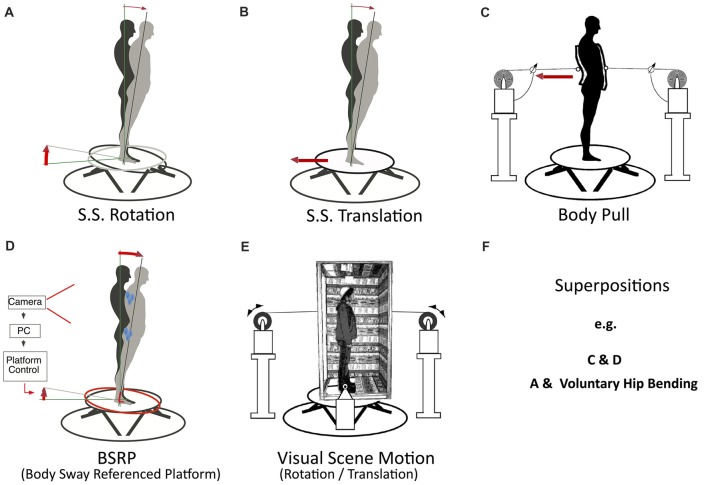
**(A–F)** Suggested posture control disturbance scenarios (inspired by human studies). Examples refer to SIP scenarios that challenge balancing of biped standing in the sagittal body plane (with moderate stimuli mainly around the ankle joints). **(A)** Support surface (SS) rotation about the ankle joint axis (using a hexapod platform; Mergner et al., 2003). **(B)** Support translation. **(C)** Contact force stimulus (applied as pull on a body harness using cable winches). **(D)** “Body sway referencing of the platform” (BSRP). Spontaneous or evoked body sway is recorded and sway signal is used to tilt the support along with the body such that the ankle joint angle (and its proprioception) remains fixed and compensation of the field force gravity with eyes closed requires vestibular input. The effect of visual self-motion and spatial orientation cues are evaluated by comparing in scenarios **(A–D)** the balancing in “eyes open” and “eyes closed” conditions. **(E)** Isolated visual scene motions, to test how successful the postural control system can suppress visually-evoked self-motion illusions (given the robot involves visual motion and orientation cues in its postural control; see text). **(F)** Combinations of two or more disturbances and of superimposing voluntary movements on external disturbances to test conflict-free interaction between proactive and reactive balancing.

The test scenarios we are suggesting for reactive balancing of *external disturbances* primarily refer to the four basic disturbances of the human posture control (compare above “Heuristic Model of Human Posture Control” and Figure 1), which equally apply to humanoid robots. Their applications are shown schematically in Figures 2A–D for the sagittal body plane, where balancing tends to be performed mainly in the ankle and hip joints: (A) *SS rotation about the ankle joints*. When using small and slow tilts in humans, disturbance torque (evoking the passive torque *T*_pass_ in Figure 1) and disturbance compensation occur foremost in the form of whole body rotation around the ankle joints, drawing on both the SS tilt compensation and the gravitational torque compensation. Large and fast tilts tend to evoke additional rotation of the upper body around the hip joints (see above; for frontal plane, see Goodworth and Peterka, 2010). These inter-link effects draw on coordination of the body segments. (B) *Support translation* (evoking *T*_in_). Similar as with tilt, responses to moderate translation stimuli involve mainly ankle joint responses, which are complemented by additional hip joint responses with rapid stimuli. The response draws specifically on the estimator of SS translation (and on the gravitational torque compensation for evoked body lean). (C) *Contact force stimulus* (evoking *T*_ext_), which draws for the postural response on the contact force estimator. The example in Figure 2C shows a controlled pull on a body harness, which typically is compensated foremost in the ankle joints. (D) Compensation of the *field force gravity* (evoking *T*_grav_) is here tested in a condition called “body sway referenced platform” (BSRP); for this test, spontaneous body sway is measured and used to tilt the SS along with the body such that the ankle joint angle (and its proprioception) remains essentially constant and balancing of upright stance with eyes closed primarily depends on vestibular input (the hip is typically not involved).

DEC in humans for the four scenarios A–D is typically performed with eyes closed vs. eyes open. The comparison allows estimating to which extent visual spatial orientation cues are used to improve non-ideal vestibular and proprioceptive disturbance estimates. Note that with vision, the secondary task of stabilizing gaze (in terms of eye-in-head and head-in-space stabilization) tends to be added to the balancing task in humans. In future, also humanoid robots may use visual motion and orientation cues to improve standing and walking balance (e.g., by using visual-vestibular fusion to improve the vestibular signals which tend to be rather noisy; compare Assländer et al., 2015). Then, a relevant test would be to evaluate standing balancing on stationary and level support in the presence of a moving scene (E). This test draws on the perceptual ability to suppress visual self-motion illusions that may result from visual surround motion (compare Mergner and Peterka, 2017).

**Figure 3:**
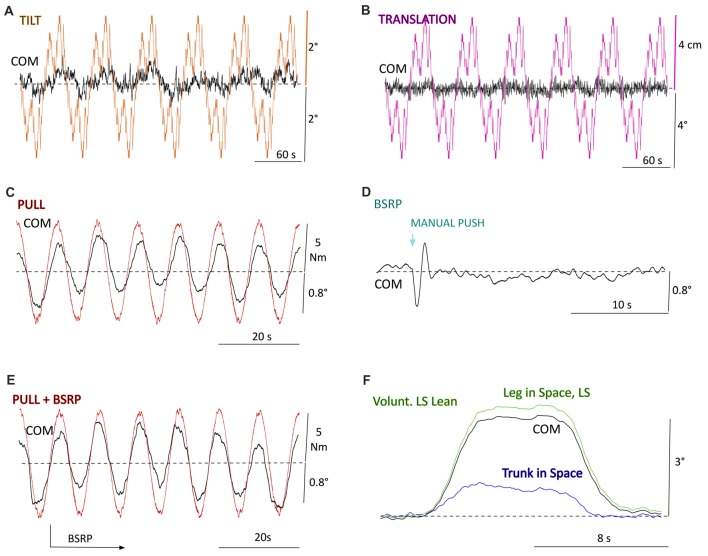
Proof of principle examples from robot experiments (Posturob II) for the disturbance scenarios **(A–D)** and **(F)** in Figure 2 (see also Table 1). **(A)** Center of mass (COM) sway responses to *pseudo-random ternary sequence stimulus* (PRTS) SS tilt of peak-to-peak (pp) 4° (six successive PRTS cycles). **(B)** Responses to horizontal SS translations (otherwise as in **A**). **(C)** COM sway responses to sinusoidal pull stimuli. **(D)** Spontaneous COM sways with “body-sway referenced platform” (BSRP) and response to a manual push perturbation. **(E)** Pull responses as in **(C)** but superimposed on BSRP (no additional push stimulus). **(F)** Commanded (“voluntary”) lean of leg segment in space (LS) around ankle joints and return to starting position with “raised cosine velocity” (RC) profiles. Associated is, as an emerging property of the DEC control, a counter-lean of trunk in space (TS) in the hip joints towards upright (dashed line), which reduces the COM excursion (TS command was to maintain trunk orientation in hip joints upright).

Not depicted in Figure 2 are scenarios of testing proactive balancing. For these, we suggest “voluntary” (commanded) whole body leans in the sagittal plane around the ankle joints (e.g., roughly sinusoidal of about 2–4° forward and back) and of the upper body around the hip joints (≈3–10° forward and back), noting corresponding compensatory counter-leans for balancing the center of mass (COM) in the hip and ankle joints, respectively (compare further below, Figure 3F; also 5A). This allows drawing on the human-inspired ability to deal, in a conflict free way, specifically with superposition of self-produced and external disturbances. To this end, we suggest superimposing such voluntary movements on concurrent SS tilts with different waveforms (Figure 2F; compare example in Figure 5B). In principle, the suggested tests (Figures 2A–C,E,F) can, in addition to the sagittal plane, also be performed in the frontal plane or some intermediate plane using the same testbed (compare Lippi and Mergner, 2017). However, interpretation is more difficult due to several factors such as the more efficient, yet also more complex body mechanics in these planes and a strong dependance on the legs’ stand-width, as demonstrated for humans (Goodworth and Peterka, 2010).

**Figure 4:**
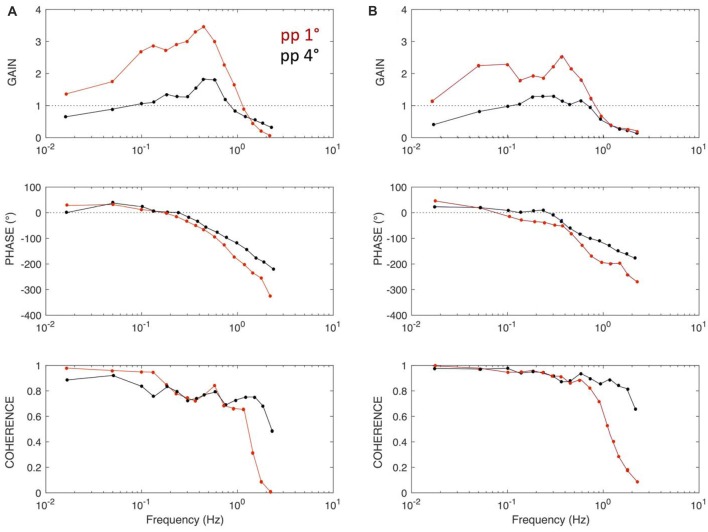
Frequency response functions (FRF) of sagittal COM sway responses for peak-peak 1° and peak-peak 4° PRTS stimuli of a human subject **(A)** and Posturob II **(B)**. Gain, phase and coherence functions across frequency characterize the tilt-evoked sway. Gain gives the amplitude ratio between sway response amplitudes and tilt stimulus amplitudes, with a gain of unity indicating that the sway response amplitude equals the stimulus sway, while a gain of zero indicates that the stimulus did not evoke any sway. Phase characterizes the temporal relation between tilt stimulus and sway response. Coherence is a measure of the signal to noise ratio of the stimulus evoked sway. Responses to this stimulus lend themselves to evaluation of human-likeness (see text).

**Figure 5:**
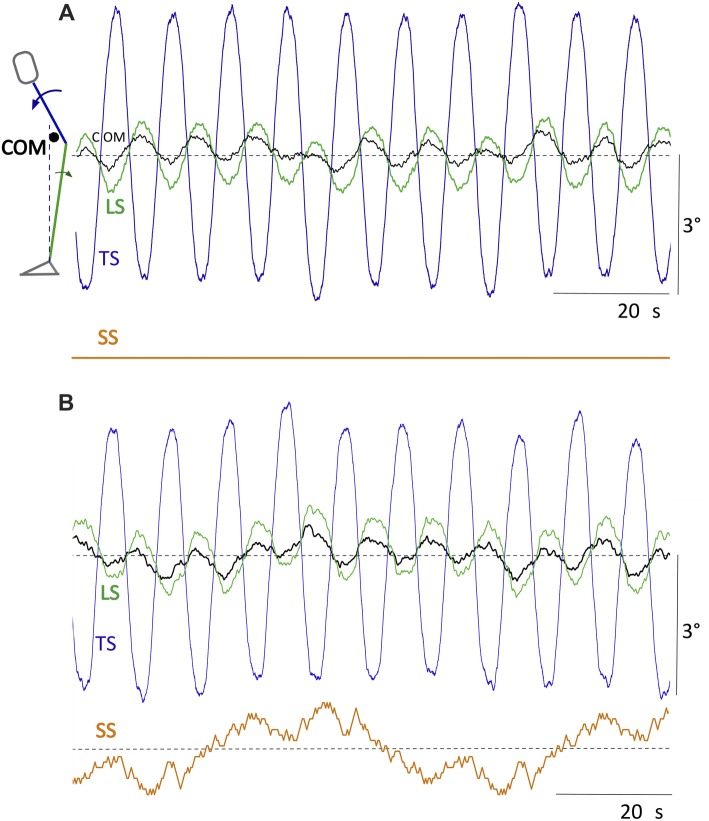
Commanded (“voluntary”) sinusoidal movements of TS of pp 3° at 0.1 Hz while standing on SS maintained level **(A)** and on SS tilting with PRTS profile **(B)**. The trunk lean movements are associated with counter leans of the LS and less so of the body COM. Command for the ankle joints was to maintain the body COM vertical.

The stimulus parameters such as stimulus magnitude and waveform may influence the usefulness of the suggested tests in robot-robot and robot-human comparisons. In human posture control, researchers nowadays typically use well-defined kinematic stimuli, most of which can equally be applied in the tests of the humanoid robot. *Sinusoidal stimuli* allow collecting responses that can be used to quantitatively characterize the response dynamics in the form of Bode diagrams. The experimental value in humans is somewhat limited, however, by the predictability of these stimuli (unless one uses sums of sine stimuli). This limits drawing conclusions about the involved sensory functions but would not be relevant in robots without implemented prediction. Humans consider prediction as difficult in the *pseudo-random ternary sequence stimulus* (PRTS), introduced by Peterka (2002; see Figures 3A,B, 4, 5B). It allows evaluation of gain, phase, and coherence of the disturbance-evoked body excursions over a defined frequency range (for data processing, see below). Transient stimuli may be applied with and without prediction (e.g., by onset announcement). A transient stimulus with “raised cosine velocity” (RC) profile is similar to a smoothed ramp and to the profile which humans use in many targeting movement tasks (the profile in the transient phase is given by *v*(t) = −*A* · *f* · cos(2π*ft*) + *A* · *f*, where *t* is time, *A* is angular displacement, and *f* is dominant frequency). Applying this stimulus with standardized parameters may allow for a fast and simple estimation of static and dynamic postural response components.

For evaluation of the postural responses in humans it often suffices for a fast overview to test the SIP scenario, and to calculate the whole-body COM responses from measures of leg and trunk excursions (recorded for example using an optoelectronic device) and the body’s anthropometrics. When the hip becomes involved, additional calculations are required to obtain the COM of the HAT (head-arms-trunk) segment. In robots, one may also calculate whole-body and HAT COM motions using internal sensor signals (e.g., from IMU and joint angle sensors). Using the PRTS stimulus requires more extensive calculations, but these have the advantage that one can obtain frequency response functions (FRFs) over a broad frequency range for different peak-to-peak (pp) amplitudes and thus can better appreciate what the hip and ankle joints contribute to the balancing in terms of dynamics (see Hettich et al., 2014).

The proposed benchmarking tests are listed in Table 1 with suggestions for stimulus magnitudes and waveforms, which we took from previous work on human balance control in our laboratory. A future aim would be to add to the table ranges for the performance measures, which are still to be established in human and robot experiments performed with a normalization for body weight and COM height (compare below). Also, considerable simplifications of the test performances may be developed and offered in future as alternatives. For example, the proposed BSRP test can be viewed as a “soft terrain” test and quantified by superimposing foam rubber layers. Overall, the suggested tests can be performed with relatively simple equipment such as a plate with an axis for tilting and a plate based on two or more roll axes for translation. Instead of the raised cosine, RC, velocity function, one may use a low-pass filtered smoothed ramp. The pull devices can be replaced by manual pulls, measuring the moment arm around the ankle joints and the pull force using a force (Newton) meter. Measures of COP require a force plate, while measures of whole-body and trunk COM requires recording of body and trunk angles respectively (for which sticks connected to potentiometers may suffice), given the body anthropometrics are known (compare Alexandrov et al., 2017). Changing weight of the body or its parts, which is known to affect human postural responses (Dietz et al., 1989), will affect also the robot’s responses, whereas preexisting weight differences between human and robots hardly affect the responses (see below).

**Table 1:** Suggested posture control benchmark tests.

	Magnitude	Body plane	Wave form	Hip fixation	Vision
(A) SS Rotation	pp 0.5° 1°, 2°, 4°	sagittal, frontal	PRTS/Sine/RC	+/−	+/−
(B) SS Translation	pp 0.5, 1, 2, 4 cm	sagittal, frontal	PRTS/Sine/RC	+/−	+/−
(C) Body pull (on pelvis/trunk)	pp 1, 2, 4, 8, 16 Nm	sagittal, frontal	PRTS/Sine/RC	−	
(D) BSRP	(Spontaneous sway)	−	−	−	+/−
(E) Visual disturbances (scene motions)	see A, B	sagittal, frontal	Sine/RC	−	−
(F) Voluntary body or trunk movements (may invoke/require inter-segmental coordination)	pp 2, 4, 8° (COM) (Rotations in ankle joints/hip joints)	sagittal, frontal	Sine/RC	−	−
(G) Recommended combinations	A & D; C & F; C & D				

*Compare Figure 2 (SS, support surface; pp, peak-to-peak; COM, center of mass; PRTS, pseudorandom ternary sequence stimulus; RC, raised cosine velocity stimulus). The following “short list” likely suffices to estimate the potential value of a more comprehensive testing: (1) RC (0.2 Hz dominant frequency) rotation of SS (compare A) with 1° and, to estimate hip cooperation, 4°. (2) 0.2 Hz RC SS translation with pp 2 cm or 4 cm displacement (see B). (3) Manual pushes against front and back of robot (compare C). (4) Instead of BSRP (D) standing on compliant support (e.g. foam rubber layers) with and without moderate pushes. (5) Commanded 2° whole body forward and backward leans in the ankle joint with RC profile. (6) Repeated 4° RC trunk forward leans (should not lead to a considerable forward lean of the leg segment). (7) Repetition of previous test 6 on SS tilting with pp 2°. The tests are restricted to the sagittal plane (no hip fixation and vision absent) and evaluation is restricted to angular motion of trunk and leg segments. Successful smooth performance with one and the same set of control parameters may be taken as human-like criterion. A corresponding long list remains to be worked out in collaboration with several robotic labs. Concerning last column, Vision, compare Figure 2 and text*.

The suggested tests draw strongly on findings for human postural responses and in robots are testing thus implicitly *human-like performance*. As already pointed out, presented with moderate external stimuli in the body’s sagittal plane, humans tend to primarily use the ankle joints for balancing stance, as if controlling an inverted pendulum (intrinsically unstable; *SIP* scenario). When increasing stimulus amplitude, especially with SS tilt stimuli, humans tend to also involve the hip joints for COM stabilization in a gradual transition from a SIP into a double inverted pendulum (DIP) balancing that uses the hip in addition. The hip contribution is known to depend on a variety of factors such as the stability of the support base or the stimulus amplitude. If insufficient disturbance compensation by the ankle joints is predicted, posture control may even primarily use the hips (McCollum et al., 1985; compare Atkeson and Stephens, 2007). In the context of reactive balancing in robot benchmarking, we consider the volitional or task/situation-dependent involvement of the hip as a human-like versatility feature and its use as fail-safe backup as the robustness feature. Interestingly, a hip-ankle coordination planning is not always required. For example, commanding the ankle joints to maintain the body COM above the ankles in a robot automatically led during “voluntary” hip bending to the “emergence” of compensatory counter-leans of the legs segment (compare Hettich et al., 2014 and below).

**Figure 6:**
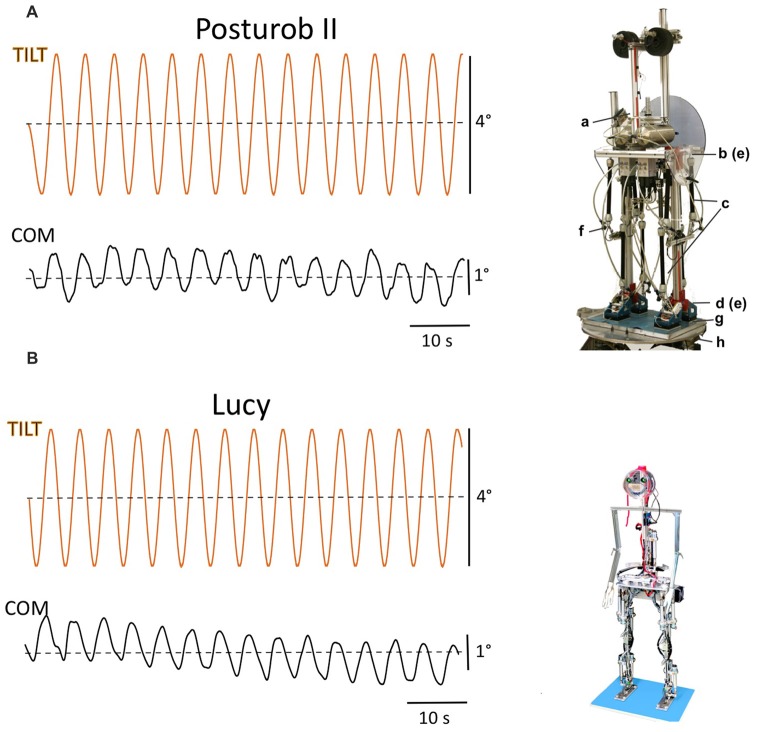
Sway responses of body COM of Posturob II **(A)** and of Lucy **(B)** to sinusoidal tilts of the SS at 0.2 Hz around the ankle joints. Note that COM response amplitudes are similar in the two experiments despite considerable differences in the robots’ anthropometrics (see text). Lower case letters in **(A)** refer to the two degree of freedom (DoF) Posturob II’s human-inspired anthropometrics, actuation, and sensors, and to the hexapod platform in the posture control laboratory: (a) artificial vestibular sensor, see Mergner et al. (2009); (b and e) hip joints with angle/angular velocity sensors; c, pneumatic muscle and f, force sensors for actuation control; (d,e) ankle joints with angle/angular velocity sensors; (g) ground reaction force sensors under heels and forefeet; (h) hexapod platform for tilt, translation, and BSRP). In the 14 DoF Lucy Posturob **(B)**, force-controlled actuation is using spindle drives; other technical features are analogous to those in Posturob II.

An important question is how to normalize the suggested balancing tests in face of the considerable differences in height, weight, and number of DoFs of the robots. Similar as in modeling approaches of human standing balance (see “Basic Aspects of Human Posture Control” section) one may treat the robot as an inverted pendulum and measure the stimulus response in terms of angular body sway. In this approach, the control of the balancing is related to the mass of the whole body COM and its height above the actuating joints (here the ankle joints, but the approach is in principle applicable also to each body segment that is held upright such as the trunk and the head with respect to its supporting joints). A further advantage is that the envisage normalization would allow to standardize the parameters used for stimulation such as the amplitude of SS tilt, for example. An example of the suggested approach is given in the “Examples of Robot Tests” section and Figure 6.

For addressing the versatility and robustness issue directly, one may inactivate the hip postural control in ankle joint tests (e.g., in the test shown in Figure 3F) and the ankle postural control in hip joint tests (test in Figures 5A,B). Finally, poor results for the here described benchmark tests may possibly predict failure in the balancing of walking, since this also involves postural control in the ankle and hip joints that respond proactively to the self-produced and reactively to unforeseen external disturbances. However, stabilizing walking balance is overall clearly more complex, involving control of body dynamics with foot placement adjustments.

## Examples of Robot Tests

Considering benchmarking of human-like versatility and robustness for humanoid robots meets already existing fruitful interrelations between the respective human and robotics research fields. For example, while robotics seeks inspiration from humans for robots (e.g., Pfeifer et al., 2007), researchers of human postural control have tested their concepts in robots for proof of principle and “real world” robustness in face of noisy and inaccurate sensors and actuation (e.g., Mergner et al., 2009; Mergner, 2010; Hettich et al., 2014; Lippi and Mergner, 2017). The tests suggested for benchmarking in Figure 2 and Table 1 have all been used in human posture control experiments and some of them also in robot experiments (see Appendix). For the envisioned benchmarking of robots, robust protocols must still be worked out to deal with the complex benchmarking issue and the large diversity of software and hardware of the robotics solutions (compare above and “Discussion” section). In the following, we present proof-of-principle examples of some of the envisioned tests. The tests were performed mainly in Posturob II, a human-inspired robot that has human-like anthropometrics, uses force controlled actuation and the DEC model (in a slightly modified form compared to Hettich et al., 2014) for postural control in two DoF (hip and ankle joints in the sagittal plane; Figures 3A–F, 4B, 5A,B, 6A; see Hettich et al., 2014, for details of the robot and its control, the optoelectronic recording of its trunk and leg motions, calculation of the body COM excursions, and the testbed, a human posture control laboratory). In all of these tests, the same set of control parameters and adjustments were used for Posturob II, and visual input was not implemented. In Figures 6A,B, tilt responses of Posturob II were compared to those of another robot, the 14 DoF robot called Lucy Posturob (anthropometrics and technical solutions are different; also Lippi and Mergner, 2017). The main results of the robot tests are listed in the following text.

Figure 3 presents responses of Posturob II as time series data for the disturbance scenarios suggested in Figures 2A–D,F. Shown are postural responses in terms of stimulus-evoked body COM sway. The examples also serve to show the three suggested stimulus waveforms (PRTS in A and B; sine waves in C and E; BSRP in D and RC for body lean in ankle joint in F). Notably, panel E shows a superposition of the pull responses (panel C) and the BSRP condition (D; push omitted in E). Note from this figure and those which follow that disturbance compensation tends to be suboptimal (considerably undershooting), which represents a typical human-likeness feature (compare “Discussion and Is Human-Likeliness an Advantage?” section).

Figure 4 shows frequency response functions (FRFs) for body COM responses to *SS tilt* in terms of gain, phase and coherence plots of a human subject (panel A; unpublished material from previous experiments conducted with written informed consent and the study was approved by the Ethics Committee of the Freiburg University Clinics and was in accordance with the 1964 Helsinki Declaration). Panel B shows corresponding responses of PostuRob II (compare the responses to the six consecutive PRTS stimuli in Figure 3A). Coherence in Figure 4 is a measure of the frequency-dependent signal-to-noise ratio (calculated by dividing the squared, absolute value of the averaged cross power spectrum by the product of the averaged input and output power spectra). Coherence is lower in the human responses than in the robot’s responses, similarly as previously reported (e.g., Hettich et al., 2011), which may suggest that humans tend to show larger response variability, attributable to larger sensor and motor noise. Otherwise the main features of the robot’s responses resemble those of the human subject. In particular, common to both is an “amplitude non-linearity” in terms of clearly larger gains of the evoked sway for the peak-to-peak (pp) 1° PRTS stimulus than for the pp = 4° stimulus (attributed to detection thresholds in the disturbance estimates; see Maurer et al., 2006; Mergner, 2010). This similarity and those for the gain and coherence curves may be used to consider a robot’s FRF more or less human-like (see below “Discussion” section).

Figure 5 shows commanded (“voluntary”) sinusoidal forward lean movements of the trunk in the sagittal body plane of Posturob II. The leg segment shows corresponding counter leans, which support the balancing of the COM over the base of support, i.e. the area under and between the feet. The counter leans emerged from the intrinsic interaction between hip and ankle DEC control modules (the command for the ankle joints was to maintain the body COM vertical above the ankles; compare Hettich et al., 2014).

Figure 6 shows a comparison between the postural responses of the robots Posturob II (A) and Lucy (B) to sinusoidal SS tilts. Body weight without feet (BW) and COM height (H) above the ankle joints of the two robots differed considerably (Posturob II: BW = 67 Kg, *H* = 167 cm; Lucy: BW = 17.5 kg, *H* = 139 cm). Note that their COM responses in terms of sway angle are, nevertheless, of similar magnitude. This owes to the fact that COM and it height are taken into account as parameters in the ankle and hip joint controllers in both robots in terms of *mgh* (where *m* gives the mass of the robot above the feet, *h* the COM height, and *g* is acceleration due to gravity; compare above, normalization across robots).

## Discussion

### Empirical Benchmarking: Quantification of Experimental Results, Metrics and Human Likeliness Measure

The experiments discussed in this article provide several possibilities to characterize the robot responses to external and self-produced disturbances. Assessing and comparing a robot’s performance require the definition of “performance metrics” on the basis of the experimental data. The first and most straightforward quantification consists in the ability to stand the imposed disturbances: for each scenario the maximum stimulus amplitude successfully tested may be used as a score. In general, however, it is not advisable to push the robots to failure. Rather, it would be desirable to obtain performance measures using basically “safe” moderate disturbances. The experiments may be then interpreted in terms of the sensitivity to the applied disturbances. For a sinusoidal stimulus, for example, gain as the ratio between the amplitude of the disturbance and the amplitude of the evoked body COM sway can be used; and, this in relation to a corresponding gain range in a human data set (to be established) may further be used as a measure of human likeness. However, this solution faces for the sinusoidal stimuli the aforementioned problem of their predictability. An alternative would be to use unpredicted “transient” stimuli with RC profile, where the uncompensated response of the system may be measured in terms of static gain (residual static lean response), overshoot and settling time. Such indices can be computed similarly for both commanded voluntary movements and external disturbances. The third possibility we consider is the PRTS stimulus. Its advantages are that humans consider it as unpredictable and that it allows for a description of the response in terms of a FRF. The total power of the FRF gives a measure of the sensitivity of the robot’s response to the applied disturbance, which can be directly compared across robots. As mentioned above, it is notable that healthy human subjects typically do not show perfect compensation of the external stimuli, but always some sway, which has been explained by some threshold mechanisms in the control. This suggests that a total disturbance rejection may overall not be an optimal solution and it certainly does not represent a human-like feature. In fact, trying to stand for some time absolutely motionless is for humans extremely difficult and soon starts to be painful.

The FRF description contains further features that may be used to quantify the human-likeness of the robot response in comparison with human data. Experiments performed with human subjects provide a behavior description that can be used for comparison with robot responses. Human behavior can be defined in terms of several features of the gain values across each represented frequency, and also by the phase and coherence. As mentioned above in the description of Figures 4A,B, such features may reveal a human-like amplitude non-linearity or, on the basis of the phase characteristics, a robustness of the control’s dynamics or, on the basis of a sufficiently high coherence, a hint on response reproducibility. It remains to be shown that such features of a robot’s FRFs can be compared with the ones observed in humans using multivariate statistical techniques. Roboticists may find it more familiar, though, to train models such as neural networks or support vector machines on the available human response datasets and classify the robots’ responses as belonging to the distributions of human responses (healthy or some particular class of patients). In both cases the idea would be to base the measure of human-likeness to reference sets of human data.

In general, we propose a set of test scenarios that relate to basic mechanisms of the human posture control system but are otherwise empirical and to a large extent independent from the particular robotic platform tested, in that the evaluation is based on measurable physical variables such as body sway and on normalizing across anthropometrics. While posture control can be considered a basic sensorimotor control skill, its efficiency also builds on lower level elements such as energy efficiency, compliance, and the actuators’ dynamic performance. These aspects can also be tested, e.g., by measuring energy consumption directly. However, such low-level issues may be too specific for the hardware used and not easily relatable to human-inspired concepts. Nevertheless, the outcomes of such tests may provide useful insights about the implemented properties. For example, in Ott et al. (2016) it is shown how including passive stiffness and delays affect posture control and balance performance. Details of the performance of the suggested tests and the evaluation protocols as well as human reference data must still be worked out.

### General Robot Evaluation Issues

In the robotics literature, different robot evaluation principles have been proposed for different tasks. In general, robots may be compared with each other with respect to their ability to solve given problems or to perform given tasks. In O’Kane and LaValle (2008), for example, evaluation of robot capabilities is inspired by the principles used in computational theory to evaluate algorithms, i.e., by evaluating whether the robot can solve a problem or not and assessing how efficiently the problem is solved. In order to perform such an evaluation, it is crucial to formally define the problem and to find measures to evaluate the efficiency of its solution. From this, one should not be misled to postulate that a benchmark framework should be specific for each robot type and task. Rather, although particular with respect to the general case (i.e., not applicable for fixed-base and wheeled robots), biped balancing is a challenge to all humanoids, and its benchmarking can be based on a set of well-defined motor tasks. Furthermore, the benchmarking can use human postural skills as a reference, which is occasionally done in human-inspired robotics, typically by evaluating a set of human skills with various levels of difficulty. For example, in the field of developmental robotics (see Guerin and Rat-Fischer, 2014) a benchmarking framework based on the skills that are progressively acquired by humans in the period from birth to early childhood is applied. Correspondingly, one may evaluate to which extent the postural responses of a humanoid resemble those of humans. Generally, the assessment of selected challenges, which are often artificial and chosen to be easily replicable, should be empirical and independent of the specific hardware implementation. Such a premise is behind the design of the Turing test in the field of artificial intelligence and its variants. Furthermore, in view that artificial agents are not (yet) able to pass the Turing test in the general sense, it has been proposed to address sub-skills that must be solved in the context and may be tested separately in order to provide at least an insight into what may be still missing (Cohen, 2005). The tests described in this article for humanoid posture control can be seen as the implementation of these principles, i.e., (1) empirical evaluation; (2) applicability to different hardware; and (3) testing sub-skills that relate to the issue of robot interaction with the real world.

### From Human Experiments to Robot Evaluation Principles

Addressing here human-like versatility and robustness for benchmarking of posture control in robots, we focused on basic components of the human posture control that are neither task-specific nor hardware-specific. These components may therefore be used to rate, predict and possibly explain the shortcomings of the sensorimotor performance of robots, or they may identify limiting amplitude and frequency margins. We conceive that apparent discrepancies between impressive task performances in internet videos and failures in robot competitions with complex “real world” scenarios owe mainly to the problems of how to cope with superposition of two or several disturbances or of active movements with external disturbances. Also, we attribute versatility and robustness of sensorimotor control to the ability of humans to exploit the multi-segment body kinematics for distributing a demanding performance across two or more joints and to use the same ability for compensating for local flaws. These features of the human posture control have basically the same importance for humanoid robots. In addition to making task performance of robots more versatile and robust, they may facilitate human-robot collaborative interactions. Our focus on human-like posture control as a crucial precondition for many movement performances is supported by a recent outline concept on benchmarking humanoid locomotion, where a considerable part is devoted to posture control (Torricelli et al., 2015).

Posture control benchmarks may improve the interpretation of general sensorimotor performance benchmarks. Low ratings for such performances may be due to either insufficient postural disturbance compensation or to suboptimal action planning, commanding, and execution control. Furthermore, postural control tests may reveal and define restrictions for one or the other external disturbance, which can then be taken into account when planning performance tasks for a robot. The overall effort and time expense for the benchmarking is larger when posture control is separately tested, but this appears acceptable when one restricts the posture control benchmarking to the very basic aspects and to a relatively small number of meaningful tests.

One may object that the described external disturbances challenge a robot’s performance especially during passive transport in a vehicle, for example. This challenge may represent an exceptional situation for which one could conceive some form of special solution, for example in terms of a passive fixation or by having the robot actively stabilizing its stance by holding with the hands. We contend, however, that challenging disturbances may have many reasons, mostly from mechanical interactions with the environment (e.g., collisions, work requiring interaction with machines or other robot agents). Furthermore, these disturbances tend also to occur during proactive movements such as walking, when body acceleration (or slowing, change in direction, etc.) produces force impacts on its buttress. The same applies to each moving body segment that is buttressing on a supporting body segment. Conceivably, focusing the suggested benchmarking tests on movements of the relatively heavy COM of the trunk and on the whole-body COM above the feet is clearly more relevant than considering head or arm movements.

The examples of robot tests shown above demonstrate that the robots’ posture control can be characterized and evaluated in terms of both body COM dynamics and inter-segmental coordination. The way in which humans exploit inter-segmental coordination depends on the functional context. For example, when a limited contact area with the SS does not allow for a sufficiently safe balancing of the body COM based on ankle torque, humans typically use additional hip movements to support the balancing, which can eventually fully take over the task. Such a “hip-strategy” emerges mainly in the presence of very intensive external perturbations (Atkeson and Stephens, 2007). In the scenarios described above, the balance behavior has not been pushed to such limits, however. In the extreme case, the hip strategy would require very forceful rapid movements that, at the current state of the art, are not implemented in robots. Such very strong perturbations may not safely be covered even by humans.

Our definition of human-like versatility and robustness and their interrelation refers in the present context to the human ability to distribute the performance of a sensorimotor task across two or more joints or to variably shift the performance from one joint to another. In the DEC concept, these mechanisms have a basis in the modular architecture of the control, including the emergence of inter-segmental movement coordination and a reduction of inter-segmental coupling forces (Hettich et al., 2014; Lippi and Mergner, 2015). Ultimately, however, the posture control mechanism as a whole builds on the ability to produce in each DoF of the skeletal system the compensation for the basic four disturbances (compare Figure 1) in a context-adequate way with respect to their overlap and timing. As already mentioned above, these responses are produced with one and the same set of control parameters and in conflict-free superposition with the movement execution control. Demonstrating this in the above robot experiments leads us to the suggestion to try the same in future robot benchmarking.

### Is Human-Likeliness an Advantage?

Referring robot benchmarking to human sensorimotor behavior and suggesting that robot performance be evaluated in terms of human-likeliness poses the general question about the value of the human-likeliness criterion. As already pointed out in “Introduction” section, human performance is still superior to that of robots and several human sensorimotor features are currently desirable for humanoids. Following this idea we proposed the evaluation of basic posture control features that allow humans to move and balance in a variety of different scenarios. Previous work (Ott et al., 2016) provides an example that using a human-inspired sensorimotor control, which includes passive joint stiffness and some form of feed-forward disturbance compensation, increases the tolerance for time delays in the control loop. Principally, the power of such a design lies in the option to use a relatively low gain in the active control; a safe choice considering the limited time margins imposed by the delay in the loop. The compliant behavior produced by the low gain, in turn, can be considered advantageous by itself for the interaction with the environment. This suggests that biological solutions may tend to address more than one problem and provide tradeoffs between different issues. On the other hand, humanoids and technical system may in principle face specific problems that are not relevant for humans (e.g., joint angles that should not hit their limits or actuators that are optimized for narrower ranges of torques and velocities compared to those of human muscles). Or, they may not be affected by the same limitations as humans (such as the long neural delays mentioned above). Because of this, the technological value of bio-inspired mechanisms depend on the specific features of the hardware involved. Nevertheless, the human-likeness feature may be of intrinsic value in human-robot interactions. Conceivably, humans would more likely perceive the motor behavior of a robot as intelligible and predictable when it is based on human-inspired control principles, which would offer safety benefits in tasks that require humans to directly collaborate with robots. This is especially important in the case of wearable robots that can partially impose motion pattern and posture control strategies to humans.

Wearable robot devices would be perceived as more transparent and reliable if they behaved in a way that reflects the natural motor schemas of the user. For this reason, we imagine that human-likeness should be evaluated specifically as one of the measures to be taken into account in a benchmarking framework for humanoids. Furthermore, several of the posture control mechanisms identified in humans may be of considerable relevance to robotic engineers. One example is given by the aforementioned solutions used by humans to deal with the relatively long *sensory feedback time delays* for disturbance estimates and the resulting challenges for control stability (compare above in “Introduction” section the inter-relation of low loop gain with low mechanical resistance and low energy consumption). Another related example, which may be potentially interesting for robotics where processing time delays are considerable, is that the human responses to the postural disturbances occur in a cascade of three steps, the first being an instant resistance from passive muscle and connective tissue properties, which is followed by an automatic and stereotype short-latency proprioceptive reflex (latency 20–40 ms), after which the context-specific multisensory and voluntarily adjustable long-latency disturbance compensation develops (often referred to as “long latency reflex”). It has been demonstrated in robots (Ott et al., 2016) that preceding the disturbance-specific counteraction by some early and fast response in equivalence to the human passive stiffness may improve control stability. A further means by which humans cope with the sensory feedback time delays is to learn external disturbances and then to predict the corresponding sensory estimates, and to also use prediction for self-produced disturbances (see “Sensory Estimations of the Four Basic Disturbances and Their Predictions” section). Preliminary robot experiments demonstrate an improvement in postural control when this includes prediction (Mergner, 2010).

Another and already previously considered point for human-likeness benchmarking is the improvement of postural control, and of sensorimotor control in general, when humans can involve visual spatial orientation and motion cues (Torricelli et al., 2016). In the absence of vision, human arm-reaches fall short, and walking slows down and becomes insecure with an increased risk of falling. Particularly strong is the beneficial effect of vision in humans with degraded vestibular function, which is consistent with the notion of a strong visual-vestibular co-operation in sensorimotor control, self-motion perception, and spatial orientation. The improvement by vision in vestibular-able subjects is mainly attributed to a reduction of high vestibular noise by the visual-vestibular signal fusion (Mergner et al., 2009; van der Kooij and Peterka, 2011; Assländer et al., 2015). A basic problem in the use of visual cues is in the evaluation of whether optic flow is stemming from self-motion or from visual surround motion. Its solution in humans involves the interpretation of a manifold of vision-derived motion and orientation cues, using cognition and learning, and it includes visual-vestibular fusion mechanisms that are still not completely understood to date (see, e.g., Mergner and Peterka, 2017). Overall, current knowledge of the human perception and sensorimotor systems is still rather limited. Studies on sensorimotor control in humans and humanoid robots will likely profit from each other and to some extent may proceed in parallel by using mutual inspirations from each other. Drawing on human-likeness inspirations for robot benchmarking to better understand, for example, the role of vision for sensorimotor skills remains a task for the future in both research fields.

## Author Contributions

TM and VL performed the experiment, collected and analyzed the data and performed the computer simulations. Both authors contributed to the interpretation of the data, drafted and critically revised the manuscript and finally approved the manuscript for submission.

## Conflict of Interest Statement

The authors declare that the research was conducted in the absence of any commercial or financial relationships that could be construed as a potential conflict of interest.

## Footnotes

^1^http://www.benchmarkinglocomotion.org/workshop-at-humanoids-2017/

^2^www.bbc.com/news/technology-33045713

## Appendix

An important further step towards benchmarking humanoids would be experiments that compare across different posture control methods in the same robot, or using the same control method across different robots (as in Figure 6). This important and necessary next step may start when a consensus is reached on the targets in the benchmarking approach. Qualitative and preliminary experiments in this direction have been performed in our laboratory. Corresponding films on posture control from our robots can be found in the internet:

(A) For Posturob I (see Mergner, 2010) under https://www.youtube.com/user/NeurologieFreiburg showing (1) Voluntary lean; (2) Balancing in response to pull stimuli when standing on foam rubber; (3) BSRP (body sway referenced platform); (4) Voluntary leans superimposed on support surface tilt (both with different frequencies); and (5) Pull stimuli applied during stance on BSRP.

(B) For similar tests using Posturob II, see https://www.youtube.com/user/neurozentrumukl Part 1: Containing balancing of tilts with (a) PRTS and (b) superimposed pushes, Part 2: Demonstrating and explaining control of voluntary movement superimposed on balancing sinusoidal tilts, Part 3: Demonstrating and explaining balancing on fixed support surface and BSRP during manual applied pushes, and Film 2: Superposition of robot and human trunk lean movements on support surface rotations.

(C) For tests in Lucy Posturob, see https://www.uniklinik-freiburg.de/neurologie/forschung/neurologische-arbeitsgruppen/postural-control/video.html demonstrating balancing of stance while performing movements simultaneously in the frontal and sagittal planes and compensating superimposed pushes.

(D) For the robot Toro from DLR using the DEC control under https://www.youtube.com/watch?v=3ALCTMW3Ei4 (see also: films referred to in Ott et al., 2016).

(E) For balancing stance of Posturob II when it used instead of the DEC an Eigenmovement control method (complementary material in Alexandrov et al., 2017).

## References

[B1] Alexandrov A. V. Lippi V. Mergner T. Frolov A. A. Hettich G. Husek D. (2017). Human-inspired eigenmovement concept provides coupling-free sensorimotor control in humanoid robot. Front. Neurorobot. 11:22. 10.3389/fnbot.2017.00022 28487646 PMC5403929

[B2] Assländer L. Hettich G. Mergner T. (2015). Visual contribution to human standing balance during support surface tilts. Hum. Mov. Sci. 41, 147–164. 10.1016/j.humov.2015.02.010 25816794 PMC4427279

[B3] Atkeson C. G. Stephens B. (2007). “Multiple balance strategies from one optimization criterion,” in Proceedings of the 7th IEEE-RAS International Conference on Humanoid Robots (Pittsburgh, PA: Institute of Electrical Engineers, IEEE), 57–64.

[B4] Bastian A. J. (1997). Mechanisms of ataxia. Phys. Ther. 77, 672–675. 10.1093/ptj/77.6.672 9184691

[B5] Cheng G. Hyon S. H. Morimoto J. Ude A. Hale J. G. Colvin G. (2007). CB: a humanoid research platform for exploring neuroscience. Adv. Robot. 21, 1097–1114. 10.1163/156855307781389356

[B6] Clever D. Mombaur K. (2017). “On the relevance of common humanoid gait generation strategies in human locomotion: an inverse optimal control approach,” in Modeling, Simulation and Optimization of Complex Processes HPSC 2015, eds Bock H. Phu H. Rannacher R. Schlöder J. (Cham: Springer), 27–40.

[B7] Cohen P. R. (2005). If not Turing’s test, then what? AI Magazine 26, 61–67. 10.1609/aimag.v26i4.1849

[B8] Dietz V. Horstmann G. A. Trippel M. Gollhofer A. (1989). Human postural reflexes and gravity—an under water simulation. Neurosci. Lett. 106, 350–355. 10.1016/0304-3940(89)90189-4 2601889

[B9] Goodworth A. D. Peterka R. J. (2010). Influence of stance width on frontal plane postural dynamics and coordination in human balance control. J. Neurophysiol. 104, 1103–1118. 10.1152/jn.00916.2009 20427616 PMC2934921

[B10] Guerin F. Rat-Fischer L. (2014). Benchmarking in developmental robotics. Available online at: http://homepages.abdn.ac.uk/f.guerin/pages/BenchmarkingChap.pdf

[B11] Hettich G. Assländer L. Gollhofer A. Mergner T. (2014). Human hip-ankle coordination emerging from multisensory feedback control. Hum. Mov. Sci. 37, 123–146. 10.1016/j.humov.2014.07.004 25150802

[B12] Hettich G. Fennell L. Mergner T. (2011). “Double inverted pendulum model of reactive human stance control,” in Multibody Dynamics Conference 2011. Available online at: https://www.uniklinik-freiburg.de/neurologie/forschung/neurologische-arbeitsgruppen/postural-control.html

[B13] Lippi V. Mergner T. (2015). “Coupling forces in human-like posture control,” in ICRA15 WS on Dynamic Locomotion and Balancing of Humanoids: State of the Art and Challenges. Available online at: https://www.uniklinik-freiburg.de/neurologie/forschung/neurologische-arbeitsgruppen/postural-control.html

[B14] Lippi V. Mergner T. (2017). Human-derived disturbance estimation and compensation (DEC) method lends itself to a modular sensorimotor control in a humanoid robot. Front. Neurorobot. 11:49. 10.3389/fnbot.2017.00049 28951719 PMC5599790

[B15] Lippi V. Mergner T. Hettich G. (2013). “A Bio-inspired modular system for humanoid posture control,” in Proceedings of IROS 2013 Workshop on Neuroscience and Robotics, eds Ugur E. Oztop E. Morimoto J. Ishii S. (Tokyo: Towards a Robot-Enabled, Neuroscience-Guided Healthy Society), 16–21.

[B16] Loeb G. E. (2012). Optimal isn’t good enough. Biol. Cybern. 106, 757–765. 10.1007/s00422-012-0514-6 22895830

[B17] Maurer C. Mergner T. Peterka R. J. (2006). Multisensory control of human upright stance. Exp. Brain Res. 171, 231–250. 10.1007/s00221-005-0256-y 16307252

[B18] McCollum G. Horak F. B. Nashner L. M. (1985). “Parsimony in neural calculations for postural movements,” in Cerebellar Functions, eds Bloedel J. R. Dichgans J. Precht W. (Berlin, Heidelberg: Springer Berlin Heidelberg), 52–66.

[B19] Mergner T. (2010). A neurological view on reactive human stance control. Ann. Rev. Control 34, 177–198. 10.1016/j.arcontrol.2010.08.001

[B22] Mergner T. Maurer C. Peterka R. J. (2003). A multisensory posture control model of human upright stance. Prog. Brain Res. 142, 189–201. 10.1016/s0079-6123(03)42014-1 12693262

[B20] Mergner T. Peterka R. J. (2017). “Human sense of balance,” in Humanoid Robotics: A Reference, eds Goswami A. Vadakkepat P. (Dordrecht: Springer), 1–38.

[B21] Mergner T. Rosemeier T. (1998). Interaction of vestibular, somatosensory and visual signals for postural control and motion perception under terrestrial and microgravity conditions—a conceptual model. Brain Res. Rev. 28, 118–135. 10.1016/s0165-0173(98)00032-0 9795180

[B23] Mergner T. Schweigart G. Fennell L. (2009). Vestibular humanoid postural control. J. Physiol. Paris 103, 178–194. 10.1016/j.jphysparis.2009.08.002 19665555

[B24] Nori F. Peters J. Padois V. Babic J. Mistry M. Ivaldi S. (2014). “Whole-body motion in humans and humanoids,” in Workshop on New Research Frontiers for Intelligent Autonomous Systems (Padova, Italy).

[B25] O’Kane J. M. LaValle S. M. (2008). Comparing the power of robots. Int. J. Robot. Res. 27, 5–23. 10.1177/0278364907082096

[B26] Ott C. Henze B. Hettich G. Seyde T. N. Roa M. A. Lippi V. (2016). Good posture, good balance: comparison of bioinspired and model-based approaches for posture control of humanoid robots. IEEE Rob. Autom. Mag. 23, 22–33. 10.1109/MRA.2015.2507098

[B27] Peterka R. J. (2002). Sensorimotor integration in human postural control. J. Neurophysiol. 88, 1097–1118. 10.1152/jn.2002.88.3.1097 12205132

[B300] Pfeifer R. Lungarella M. Iida F. (2007). Self-organization, embodiment, and biologically inspired robotics. Science 318, 1088–1093. 10.1126/science.1145803 18006736

[B29] Torricelli D. Gonzalez-Vargas J. Veneman J. F. Mombaur K. Tsagarakis N. del-Ama A. J. (2015). Benchmarking bipedal locomotion: a unified scheme for humanoids, wearable robots and humans. IEEE Rob. Autom. Mag. 22, 103–115. 10.1109/MRA.2015.2448278

[B28] Torricelli D. Gonzalez J. Weckx M. Jiménez-Fabián R. Vanderborght B. Sartori M.. (2016). Human-like compliant locomotion: state of the art of robotic implementations. Bioinspir. Biomim. 11:051002. 10.1088/1748-3190/11/5/051002 27545108

[B30] van der Kooij H. Peterka R. J. (2011). Non-linear stimulus-response behavior of the human stance control system is predicted by optimization of a system with sensory and motor noise. J. Comput. Neurosci. 30, 759–778. 10.1007/s10827-010-0291-y 21161357 PMC3108015

[B31] Visser J. E. Bloem B. R. (2005). Role of the basal ganglia in balance control. Neural Plast. 12, 161–174. 10.1155/np.2005.161 16097484 PMC2565457

[B32] Vukobratović M. Borovac B. (2004). Zero-moment point—thirty five years of its life. Int. J. Hum. Robot. 1, 157–173. 10.1142/s0219843604000083

[B33] Wolpert D. M. Flanagan J. R. (2001). Motor prediction. Curr. Biol. 11, R729–R732. 10.1016/S0960-9822(01)00432-8 11566114

[B34] Zebenay M. Lippi V. Mergner T. (2015). “Human-like humanoid robot posture control,” in Proceedings of the 12th International Conference on Informatics in Control, Automation and Robotics (ICINCO) (Colmar, France), 304–309.

